# Supplementary material for: Particle Engineering of Innovative Nanoemulsion Designs to Modify the Accumulation in Female Sex Organs by Particle Size and Surface Charge
Source: Pharmaceutics. 2022 Jan 27;14(2):301. doi: 10.3390/pharmaceutics14020301 (PMC8877295; doi:10.3390/pharmaceutics14020301)
Supplement: Supplementary file 1 [file pharmaceutics-14-00301-s001.zip › pharmaceutics-1469590-supplementary.pdf]

pharmaceutics

MDPI

Supplementary Materials: Particle Engineering of Innovative Nanoemulsion Designs to Modify the Accumulation in Female Sex Organs by Particle Size and Surface Charge

Eike Folker Busmann and Henrike Lucas

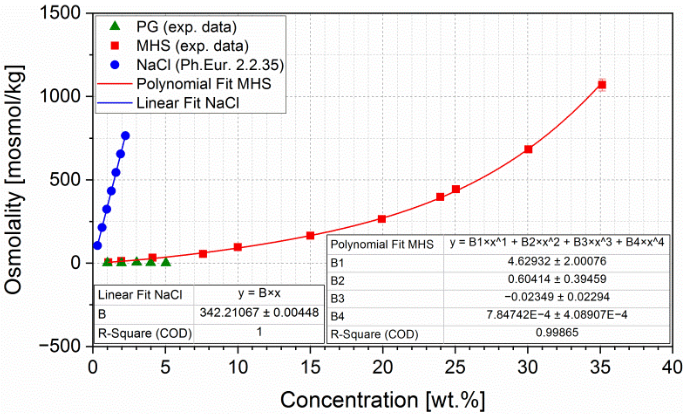

Figure S1. The ideal osmolality of aqueous sodium chloride solutions after Ph.Eur. 2.2.35 and experimental (exp.) determined osmolality of aqueous dissolved MHS and PG.

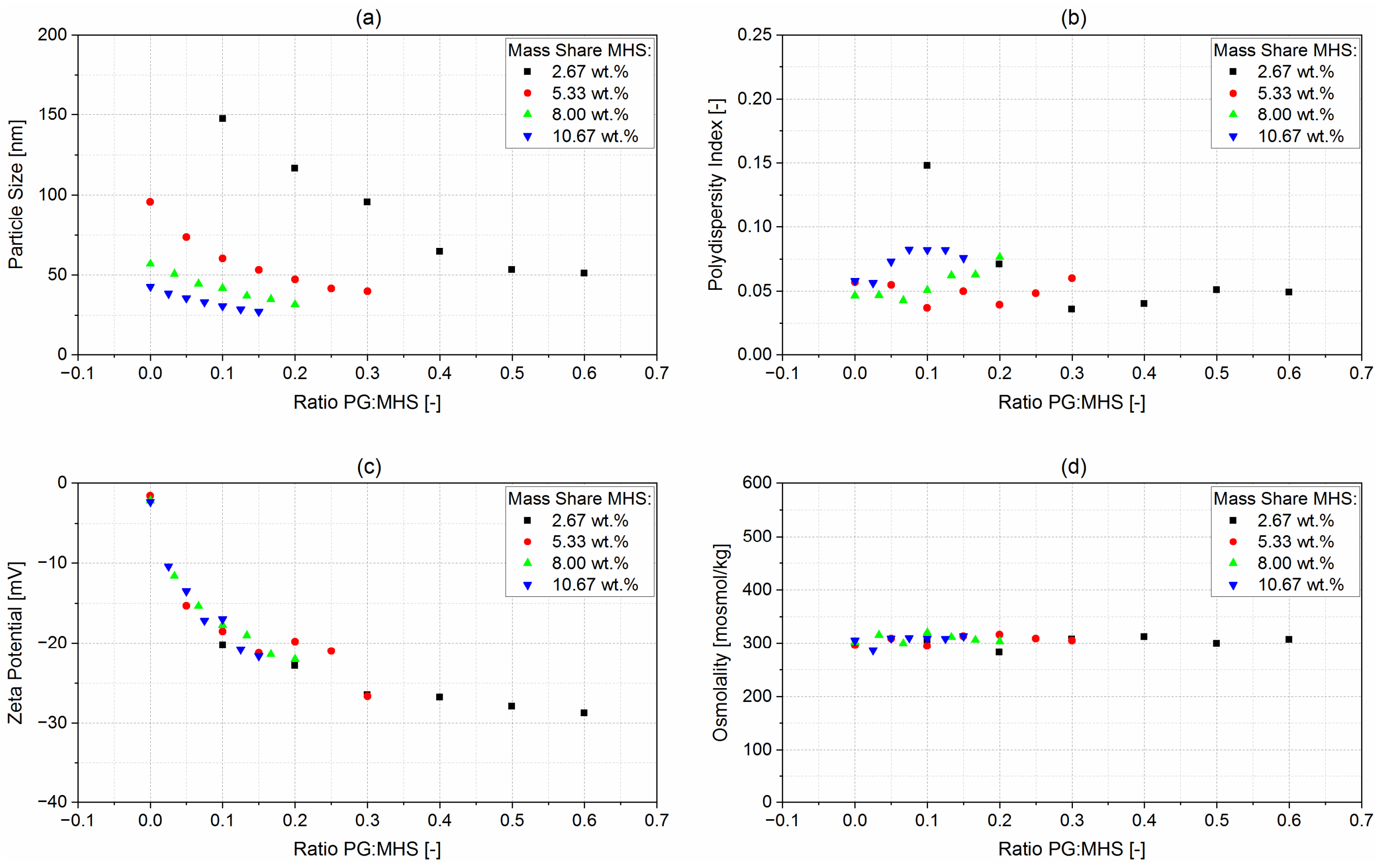

Figure S2. Scatter plots showing (**a**) the resulting particle diameter, (**b**) the PDI, (**c**) the zeta potential in 0.1x PBS at pH 7.4, and (**d**) the osmolality of the screening trials against the PG:MHS ratio at constant MCT mass share of 8 wt.%.

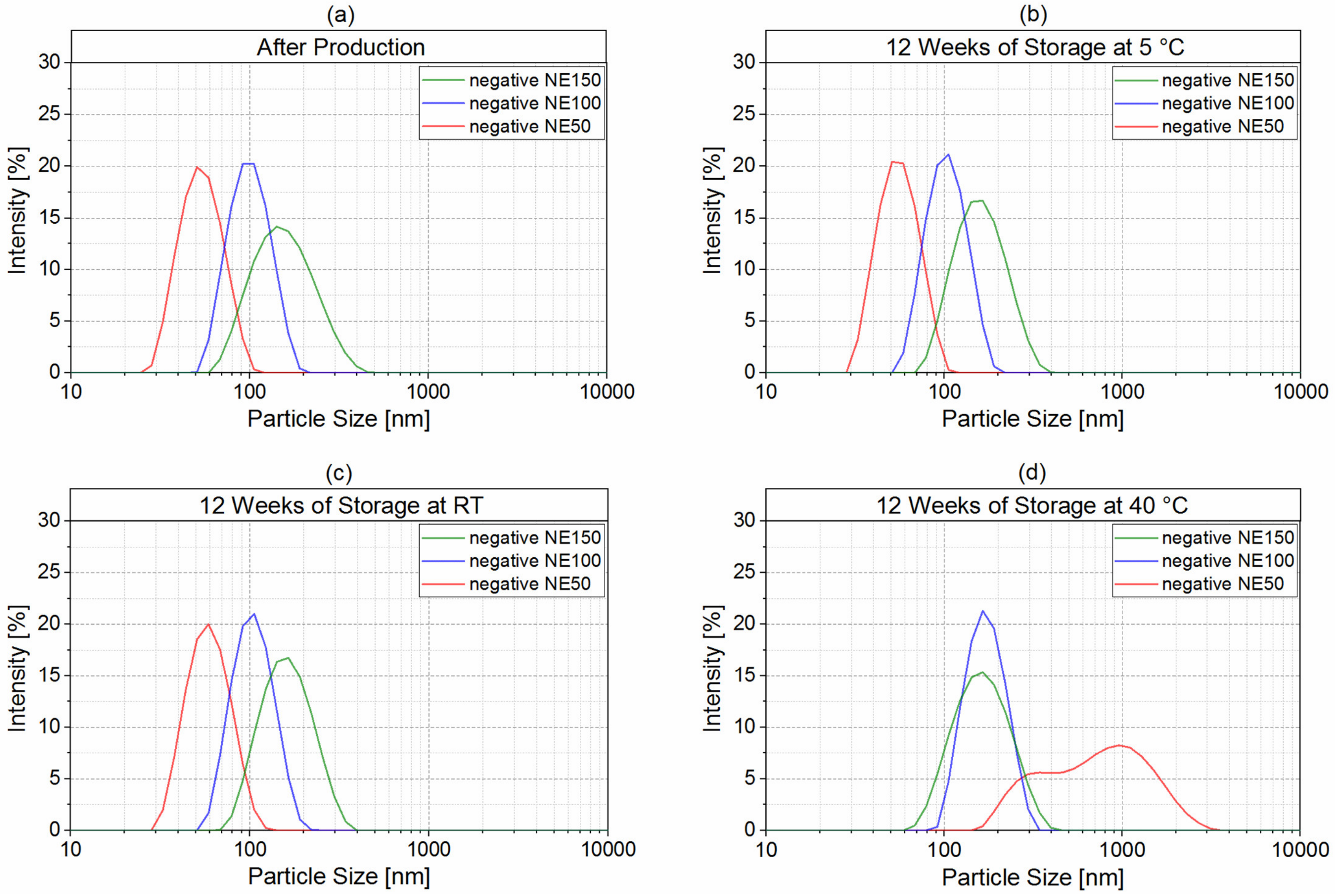

Figure S3. Particle size distributions of the negatively surface-charged nanoemulsions (**a**) after production and after 12 weeks of storage at the recommended storage conditions according to the ICH guidelines Q1A of (**b**) 5 ± 3 °C, (**c**) room temperature (RT), and (**d**) 40 ± 2 °C.

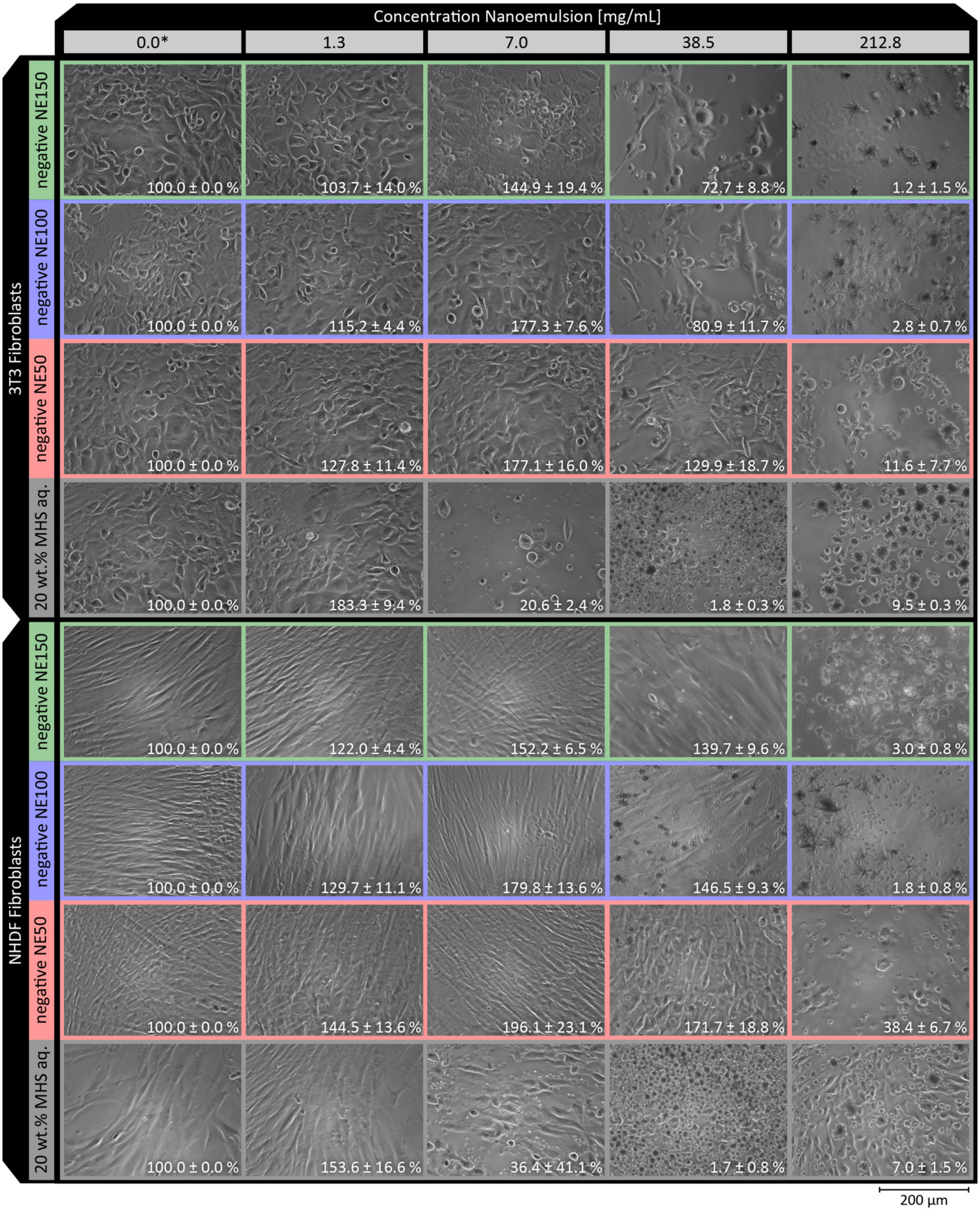

Concentration Nanoemulsion [mg/mL]
0.0*
1.3
7.0
38.5
212.8
3T3 Fibroblasts
negative NE150
100.0 ± 0.0 %
103.7 ± 14.0 %
144.9 ± 19.4 %
72.7 ± 8.8 %
1.2 ± 1.5 %
negative NE100
100.0 ± 0.0 %
115.2 ± 4.4 %
177.3 ± 7.6 %
80.9 ± 11.7 %
2.8 ± 0.7 %
negative NE50
100.0 ± 0.0 %
127.8 ± 11.4 %
177.1 ± 16.0 %
129.9 ± 18.7 %
11.6 ± 7.7 %
20 wt.% MHS aq.
100.0 ± 0.0 %
183.3 ± 9.4 %
20.6 ± 2.4 %
1.8 ± 0.3 %
9.5 ± 0.3 %
NHDF Fibroblasts
negative NE150
100.0 ± 0.0 %
122.0 ± 4.4 %
152.2 ± 6.5 %
139.7 ± 9.6 %
3.0 ± 0.8 %
negative NE100
100.0 ± 0.0 %
129.7 ± 11.1 %
179.8 ± 13.6 %
146.5 ± 9.3 %
1.8 ± 0.8 %
negative NE50
100.0 ± 0.0 %
144.5 ± 13.6 %
196.1 ± 23.1 %
171.7 ± 18.8 %
38.4 ± 6.7 %
20 wt.% MHS aq.
100.0 ± 0.0 %
153.6 ± 16.6 %
36.4 ± 41.1 %
1.7 ± 0.8 %
7.0 ± 1.5 %
200 µm

Figure S4. Phase contrast microscopy images at 20x magnification showing the morphology changes of the 3T3 and NHDF fibro-blasts with increasing nanoemulsion concentration including the formation of needle-like 12 hydroxystearic acid crystals after 24 h incubation with the negatively surface charged nanoemulsions NE50, NE100, NE150, and a 20 wt.% MHS aqueous (aq.) solution (*negative control, mean cell viability ± standard deviation given as a percentage in the microscopy images).

Table S1. Mean PARE values plus standard deviation (*n* = 5) of each excised organ plus withdrawn blood of the adult mice i.v. injected with the negative NE50, NE100, or NE150 nanoemulsions and additionally previously investigated neutral NE25, NE50, NE100, or NE150 nanoemulsions.

Mean PARE [%] of the Excised Organs	Negative NE50	Negative NE100	Negative NE150	Neutral NE25	Neutral NE50	Neutral NE100	Neutral NE150
blood	0.9 ± 0.4	0.7 ± 0.1	0.7 ± 0.1	0.3 ± 0.1	0.4 ± 0.1	0.8 ± 0.2	0.9 ± 0.3
heart	3.2 ± 0.6	5.9 ± 1.3	8.1 ± 1.5	2.1 ± 0.5	2.1 ± 0.4	4.1 ± 0.8	5.9 ± 0.6
lungs	3.0 ± 0.1	2.5 ±1.1	3.0 ± 2.2	2.6 ± 0.4	1.6 ± 0.2	1.8 ± 0.3	2.1 ± 0.3
liver	51.5 ± 0.5	48.3 ± 1.8	42.1 ± 2.4	66.2 ± 1.6	62.8 ± 2.6	46.4 ± 4.7	40.3 ± 3.6
kidneys	3.3 ± 0.3	4.8 ± 0.5	5.0 ± 0.8	2.4 ± 0.3	3.0 ± 0.6	4.2 ± 0.4	4.6 ± 0.2
bladder	1.7 ± 0.6	1.0 ± 0.2	1.0 ± 0.3	0.6 ± 0.1	0.7 ± 0.2	1.2 ± 0.2	1.1 ± 0.1
spleen	8.9 ± 1.4	8.2 ± 1.1	9.9 ± 1.0	6.7 ± 0.8	11.4 ± 1.0	12.9 ± 1.2	8.3 ± 0.9
pancreas + fat	1.8 ± 0.3	1.7 ± 0.3	2.5 ± 0.6	0.8 ± 0.2	0.9 ± 0.1	1.4 ± 0.3	1.7 ± 0.2
stomach	5.7 ± 1.5	4.7 ± 0.3	7.7 ± 0.9	2.0 ± 0.6	2.0 ± 0.5	4.3 ± 0.5	6.4 ± 1.2
duodenum	2.2 ± 0.5	1.5 ± 0.3	1.8 ± 0.2	1.4 ± 0.2	1.2 ± 0.3	1.7 ± 0.3	2.1 ± 0.4
caecum	3.7 ± 0.5	4.3 ± 0.9	3.5 ± 0.6	1.4 ± 0.6	1.7 ± 0.4	3.1 ± 1.1	4.7 ± 0.3
colon	1.0 ± 0.1	1.0 ± 0.1	0.9 ± 0.2	0.6 ± 0.2	0.5 ± 0.2	1.6 ± 1.6	1.0 ± 0.1
brain	0.6 ± 0.1	1.4 ± 1.4	0.7 ± 0.1	0.5 ± 0.0	0.4 ± 0.1	0.6 ± 0.1	0.8 ± 0.1
skin	2.0 ± 0.3	1.8 ± 0.2	1.6 ± 0.5	0.8 ± 0.1	1.6 ± 0.2	2.5 ± 0.8	3.3 ± 0.7
s.c. fat	1.5 ± 0.9	2.8 ± 1.4	2.0 ± 0.6	1.6 ± 1.3	1.2 ± 0.3	1.5 ± 0.4	2.3 ± 0.3
femur, knee + tibia	3.0 ± 0.5	3.3 ± 0.4	2.9 ± 0.2	3.4 ± 0.8	4.3 ± 0.6	4.6 ± 0.5	3.3 ± 0.4
thigh muscle	0.9 ± 0.1	1.4 ± 0.3	1.6 ± 0.5	1.5 ± 1.2	0.6 ± 0.1	1.2 ± 0.3	1.4 ± 0.2
uterus + ovaries	5.1 ±1.6	4.8 ± 0.7	4.9 ± 1.0	5.2 ± 1.6	3.5 ± 0.4	5.9 ± 2.8	9.8 ± 2.3

Table S2. Calculated 2-sided exact significance p and corresponding U value of the PAREs with the three negatively surface-charged nanoemulsions by MWU, showing the effects of the particle size onto the PAREs.

	**Negative NE50 and Negative NE100**		**Negative NE50 and Negative NE150**		**Negative NE100 and Negative NE150**	
	**p [-]**	**U [-]**	**p [-]**	**U [-]**	**p [-]**	**U [-]**
blood	0.960	10.0	0.421	8.0	1.000	12.0
heart	0.008**	0.0	0.008**	0.0	0.056	3.0
lungs	0.151	5.0	0.151	5.0	0.690	10.0
liver	0.008**	0.0	0.008**	0.0	0.016*	1.0
kidneys	0.008**	0.0	0.008**	0.0	1.000	12.0
bladder	0.032*	2.0	0.056	3.0	1.000	12.0
spleen	0.222	6.0	0.310	7.0	0.056	3.0
pancreas + fat	0.548	9.0	0.032*	2.0	.008**	0.0
stomach	0.151	5.0	0.032*	2.0	.008**	0.0
duodenum	0.032*	2.0	0.222	6.0	0.056	3.0
caecum	0.310	7.0	1.000	12.0	0.151	5.0
colon	0.841	11.0	0.310	7.0	0.690	10.0
brain	0.095	4.0	0.421	8.0	0.016*	1.0
skin	0.421	8.0	0.095	4.0	0.690	10.0
s.c. fat	0.095	4.0	0.222	6.0	0.421	8.0
femur, knee + tibia	0.310	7.0	1.000	12.0	0.151	5.0
thigh muscle	0.016*	1.0	0.008**	0.0	0.690	10.0
uterus + ovaries	0.310	7.0	0.548	9.0	0.841	11.0

Table S3. Calculated 2-sided exact significance p and corresponding U value of the PAREs with the four neutral surface-charged nanoemulsions by MWU, showing the effects of the particle size onto the PAREs.

	**Neutral NE25 and Neutral NE50**		**Neutral NE25 and Neutral NE100**		**Neutral NE25 and Neutral NE150**		**Neutral NE50 and Neutral NE100**		**Neutral NE50 and Neutral NE150**		**Neutral NE100 and Neutral NE150**	
	**p [-]**	**U [-]**	**p [-]**	**U [-]**	**p [-]**	**U [-]**	**p [-]**	**U [-]**	**p [-]**	**U [-]**	**p [-]**	**U [-]**
blood	0.095	4.0	0.008**	0.0	0.008**	0.0	0.016*	1.0	0.016*	1.0	0.421	8.0
heart	1.000	12.0	0.008**	0.0	0.008**	0.0	0.008**	0.0	0.008**	0.0	0.016*	1.0
lungs	0.008**	0.0	0.016*	1.0	0.095	4.0	0.421	8.0	0.016*	1.0	0.151	5.0
liver	0.032*	2.0	0.008**	0.0	0.008**	0.0	0.008**	0.0	0.008**	0.0	0.032*	2.0
kidneys	0.095	4.0	0.008**	0.0	0.008**	0.0	0.008**	0.0	0.008**	0.0	0.151	5.0
bladder	0.548	9.0	0.008**	0.0	0.008**	0.0	0.008**	0.0	0.008**	0.0	0.841	11.0
spleen	0.008**	0.0	0.008**	0.0	0.008**	0.0	0.056	3.0	0.008**	0.0	0.008**	0.0
pancreas + fat	0.690	10.0	0.008**	0.0	0.008**	0.0	0.008**	0.0	0.008**	0.0	0.095	4.0
stomach	0.841	11.0	0.008**	0.0	0.008**	0.0	0.008**	0.0	0.008**	0.0	0.008**	0.0
duodenum	0.421	8.0	0.095	4.0	0.016*	1.0	0.056	3.0	0.016*	1.0	0.095	4.0
caecum	0.151	5.0	0.032*	2.0	0.008**	0.0	0.032*	2.0	0.008**	0.0	0.008**	0.0
colon	1.000	12.0	0.032*	2.0	0.008**	0.0	0.032*	2.0	0.008**	0.0	0.548	9.0
brain	0.008**	0.0	0.056	3.0	0.008**	0.0	0.008**	0.0	0.008**	0.0	0.008**	0.0
skin	0.008**	0.0	0.008**	0.0	0.008**	0.0	0.151	5.0	0.008**	0.0	0.222	6.0
s.c. fat	0.690	10.0	0.310	7.0	0.151	5.0	0.310	7.0	0.008**	0.0	0.032*	2.0
femur, knee + tibia	0.095	4.0	0.032*	2.0	1.000	12.0	0.548	9.0	0.016*	1.0	0.008**	0.0
thigh muscle	0.841	11.0	0.690	10.0	0.690	10.0	0.008**	0.0	0.008**	0.0	0.548	9.0
uterus + ovaries	0.056	3.0	0.548	9.0	0.008**	0.0	0.151	5.0	0.008**	0.0	0.095	4.0

Table S4. Calculated 2-sided exact significance p and corresponding U value of the PAREs with similar-sized nanoemulsions with negative and neutral surface charge by MWU, showing the effects of the surface charge onto the PAREs.

	Negative NE50 and Neutral NE50		Negative NE100 and Neutral NE100		Negative NE150 and Neutral NE150	
	**p [-]**	**U [-]**	**p [-]**	**U [-]**	**p [-]**	**U [-]**
blood	0.032*	2.0	0.690	10.0	0.548	9.0
heart	0.056	3.0	0.032*	2.0	0.056	3.0
lungs	0.008**	0.0	0.421	8.0	1.000	12.0
liver	0.008**	0.0	0.222	6.0	0.841	11.0
kidneys	0.690	10.0	0.056	3.0	0.548	9.0
bladder	0.008**	0.0	0.310	7.0	0.151	5.0
spleen	0.032*	2.0	0.008*	0.0	0.032*	2.0
pancreas + fat	0.008**	0.0	0.151	5.0	0.008**	0.0
stomach	0.008**	0.0	0.222	6.0	0.095	4.0
duodenum	0.008**	0.0	0.222	6.0	0.310	7.0
caecum	0.008**	0.0	0.151	5.0	0.016*	1.0
colon	0.008**	0.0	0.690	10.0	0.310	7.0
brain	0.008**	0.0	0.008**	0.0	0.032*	2.0
skin	0.095	4.0	0.151	5.0	0.008**	0.0
s.c. fat	1.000	12.0	0.095	4.0	0.548	9.0
femur, knee + tibia	0.008**	0.0	0.008**	0.0	0.151	5.0
thigh muscle	0.008**	0.0	0.421	8.0	0.690	10.0
uterus + ovaries	0.151	5.0	0.548	9.0	0.008**	0.0
